# Neuregulin-4 attenuates diabetic cardiomyopathy by regulating autophagy via the AMPK/mTOR signalling pathway

**DOI:** 10.1186/s12933-022-01643-0

**Published:** 2022-10-11

**Authors:** Hongchao Wang, Lijie Wang, Fuli Hu, Pengfei Wang, Yanan Xie, Fang Li, Bingyan Guo

**Affiliations:** 1 Department of Cardiovascular Medicine, The Second Hospital of Hebei Medical University, Heping West Road No. 215, Shijiazhuang, 050000 China; 2 Department of Cardiology, Shijiazhuang Great Wall Hospital of Integrated Traditional Chinese and Western Medicine, Shijiazhuang, 050000 China; 3 Hebei Key Laboratory of Laboratory Medicine, The Second Hospital of Hebei Medical University, Shijiazhuang, 050000 China

**Keywords:** Neuregulin-4, Diabetic cardiomyopathy, Autophagy, Signalling pathway

## Abstract

**Background:**

Diabetic cardiomyopathy is characterized by left ventricle dysfunction, cardiomyocyte apoptosis, and interstitial fibrosis and is a serious complication of diabetes mellitus (DM). Autophagy is a mechanism that is essential for maintaining normal heart morphology and function, and its dysregulation can produce pathological effects on diabetic hearts. Neuregulin-4 (Nrg4) is an adipokine that exerts protective effects against metabolic disorders and insulin resistance. The aim of this study was to explore whether Nrg4 could ameliorate DM-induced myocardial injury by regulating autophagy.

**Methods:**

Four weeks after the establishment of a model of type 1 diabetes in mice, the mice received Nrg4 treatment (with or without an autophagy inhibitor) for another 4 weeks. The cardiac functions, histological structures and cardiomyocyte apoptosis were investigated. Autophagy-related protein levels along with related signalling pathways that regulate autophagy were evaluated. In addition, the effects of Nrg4 on autophagy were also determined in cultured primary cardiomyocytes.

**Results:**

Nrg4 alleviated myocardial injury both in vivo and in vitro. The autophagy level was decreased in type 1 diabetic mice, and Nrg4 intervention reactivated autophagy. Furthermore, Nrg4 intervention was found to activate autophagy via the AMPK/mTOR signalling pathway. Moreover, when autophagy was suppressed or the AMPK/mTOR pathway was inhibited, the beneficial effects of Nrg4 were diminished.

**Conclusion:**

Nrg4 intervention attenuated diabetic cardiomyopathy by promoting autophagy in type 1 diabetic mice. Additionally, Nrg4 induced autophagy via the AMPK/mTOR signalling pathway.

**Supplementary Information:**

The online version contains supplementary material available at 10.1186/s12933-022-01643-0.

## Background

Heart failure is a major cardiac complication that contributes to the increased morbidity and mortality of diabetes mellitus (DM) [1]. Emerging evidence has revealed that nearly a quarter of patients with diabetes eventually develop heart failure [2]. Diabetic cardiomyopathy is a myocardial-specific complication that is associated with coronary microvascular dysfunction and increases the risk of heart failure in patients with diabetes [3]. Diabetic cardiomyopathy is characterized by a series of structural and functional abnormities, including left ventricular dysfunction, cardiomyocyte apoptosis, and interstitial fibrosis, all of which develop in the absence of coronary artery disease, valvular disease, and hypertension [4, 5]. Therefore, the pathological mechanisms of and effective treatments for diabetic cardiomyopathy need to be explored further [6].

Neuregulin-4 (Nrg4) is primarily produced by brown adipose tissue and belongs to the neuregulin family of epidermal growth factors. It has important functions in cellular proliferation, migration and differentiation [7, 8]. Accumulating evidence has demonstrated that Nrg4 exerts beneficial effects on glycolipid metabolism in animal models. Such effects include promoting fuel oxidation and reducing adiposity, stimulating the basal metabolic rate, improving systemic metabolism [9], and playing protective roles against diet-induced insulin resistance [10–12]. Moreover, clinical studies have also found that Nrg4 is associated with metabolic disorders. Most of these studies indicated that reduced serum levels of Nrg4 may play a substantial role in the development of diabetes [13–16]. Despite these findings, little is known about the molecular mechanisms by which Nrg4 acts in DM.

Autophagy is an evolutionarily conserved quality-control system that delivers damaged organelles and cytoplasmic components to lysosomes for degradation and recirculation. This process plays a critical role in maintaining normal intracellular homoeostasis [17]. It is generally believed that autophagy is crucial to maintaining normal heart morphology and function [18]. However, the precise role of autophagy in diabetic cardiomyopathy has yet to be clearly deciphered. Both the suppression and hyperactivation of cardiac autophagy can have a pathological impact on diabetic hearts. Autophagy dysregulation has been observed in diabetic hearts in several preclinical studies [19–21]. Thus, autophagy regulation could represent a potential pharmacotherapeutic strategy for diabetes-provoked cardiac ailments.

The upstream signalling pathways that regulate autophagy have been extensively explored. Accumulating evidence has revealed that the AMPK/mTOR pathway plays a key role in the autophagy regulation of diabetic cardiomyopathy [22, 23]. Targeting this signalling pathway is considered a potential therapeutic approach for diabetic cardiomyopathy [24]. Emerging evidence has demonstrated that Nrg4 can activate autophagy through the AMPK/mTOR signalling pathway to alleviate hepatic steatosis [25]. Therefore, we speculated that Nrg4 could attenuate diabetic cardiomyopathy by regulating autophagy and explored the underlying molecular signalling pathway using a type 1 diabetes mouse model.

## Methods

### Experimental animals

Male C57BL/6J mice of 8 weeks of age were obtained from SPF Biotechnology Co., Ltd. (Beijing, China). All mice were housed in standard conditions (temperature 22 ± 1 °C and 12 h light/dark cycle) with free access to a standard rodent diet and tap water. The experiment was approved by the Research Ethics Committee of the Second Hospital of Hebei Medical University and was performed in accordance with the Principles of Welfare of Experiment Animal formulated by Hebei Medical University.

### Model establishment and experimental groups

The type 1 diabetes model was established by intraperitoneal injections of 50 mg/kg freshly prepared streptozotocin (STZ; Sigma, St. Louis, MO, USA) dissolved in 0.1 mM sodium citrate buffer (pH 4.5) for 5 consecutive days. The control mice received the same volume of sodium citrate buffer. One week after the injections, tail vein blood was randomly collected. The glucose values were ≥ 16.7 mmol/L, as measured by a glucometer (Accu-Chek, Roche Diagnostics, Basel, Switzerland). These levels confirmed the successful establishment of the model [26].

Animal experiments were performed in two steps. The first study investigated the protective effect of Nrg4 on the diabetic heart and its putative mechanism in autophagy. Mice were randomly divided into four groups (n = 6 per group): the nondiabetic control (CON) group, the type 1 diabetic mouse (DM) group, the type 1 diabetic mouse with Nrg4 treatment (DM + Nrg4) group, and the nondiabetic mouse with Nrg4 treatment (CON + Nrg4) group. Four weeks after successful modelling, the DM + Nrg4 group and CON + Nrg4 group received intraperitoneal injections of recombinant Nrg4 (Sino Biological Inc, Beijing, China) for another 4 weeks. The dose (100 µg/kg, three times per week) was based on the results of previous studies [25, 27]. The CON group and DM group were given the same volume of vehicle (PBS). Next, cardiac function was measured. Then, animals were euthanized, and heart tissues were collected. The next study was based on the results of the first step, and autophagy inhibitors were added to further confirm the mechanism by which Nrg4 acts. There were four groups (n = 6 per group): the DM, DM + Nrg4, DM + Nrg4 + 3MA and DM + 3MA groups. For the DM + Nrg4 and DM + Nrg4 + 3MA groups, Nrg4 was used in the same way as described above, and the DM and DM + 3MA groups were given the same volume of vehicle. The autophagy inhibitor 3-methyladenine (3-MA; MCE, Monmouth Junction, NJ, USA) was applied (30 mg/kg, intraperitoneal, three times per week) for 2 weeks before the end of the study in the DM + Nrg4 + 3MA and DM + 3MA groups [28], while intraperitoneal injections of vehicle (PBS) were administered in the DM and DM + Nrg4 groups three times a week. The duration of the second step was also 8 weeks.

### Cell culture

Primary cardiomyocytes were isolated from newborn (1 to 3-day-old) C57BL/6J mice according to previous methods [29, 30]. These cells were cultured in Dulbecco’s modified Eagle’s medium (DMEM) supplemented with 10% foetal bovine serum and 1% penicillin/streptomycin in a 95% O_2_ and 5% CO_2_ incubator at 37 °C. When the cardiomyocyte populations reached 50–60% confluence, they were exposed to either normal glucose (NG) or high glucose (HG) culture medium. HG culture medium was made using DMEM complete medium (5.5 mM glucose) supplemented with D-glucose to a final concentration of 30 mM [22]. The NG medium was made using DMEM (5.5 mM glucose) supplemented with 24.5 mM mannitol. The cells were pretreated with HG or NG for 48 h and then incubated with Nrg4 (100 ng/ml) in the absence or presence of 3-MA (2 mM) [30] or compound C (10 µM, Comp C; MCE) [22, 31] for another 12 h, according to a previous study [25].

### Echocardiography

High-resolution transthoracic echocardiography (Vevo LAB, VisualSonics, Toronto, ON, Canada) was performed on mice anaesthetized with Avertin. Two-dimensional guided M-mode measurements of the left ventricular inner diameter were obtained from the short-axis view at the level of the papillary muscle for at least 3 beats and averaged. The left ventricular end-diastolic diameter (LVEDD) and end-systolic diameter (LVESD) were measured. The left ventricular end-diastolic volume (LVEDV), left ventricular end-systolic volume (LVESV), left ventricular ejection fraction (LVEF) and left ventricular fractional shortening (LVFS) were calculated by computer algorithms. The parameters are calculated [32] as follows: V = [7.0 / (2.4 + D)] × D^3^; LVEF = (LVEDV - LVESV)/LVEDV × 100%; LVFS = (LVEDD - LVESD)/LVEDD × 100%.

### Histological staining

The heart samples were fixed in 4% paraformaldehyde, processed via dehydration, embedded in paraffin and sliced into 5 μm thick sections. Haematoxylin & eosin (HE) staining and Masson’s trichrome staining were performed. The morphological changes and collagen content of the cardiac tissues were observed with an optical microscope. ImageJ software was employed to measure the collagen area in 5 fields from each imaged section and to analyse the collagen volume fraction (CVF). CVF = average collagen area/area of total field × 100%.

### Transmission electron microscopy (TEM)

First, the primary cardiomyocytes were fixed with 4% glutaraldehyde. Next, they were postfixed with osmium tetroxide (1%) and dehydrated with a graded series of ethanol (70%, 90%, and 100%). Finally, the samples were embedded and cut into ultrathin sections (70–80 nm). These sections were examined with an electron microscope operating at 80 kV.

### Western blot analysis

Proteins were obtained from the hearts or cell extracts and were analysed using Western blot analysis, as previously described [33]. The following primary antibodies were used: anti-LC3 (1:500; Abcam, Cambridge, MA, UK), anti-p62 (1:500; Abcam), anti-Beclin1 (1:500; Abcam), anti-AMPK (1:1000; Cell Signaling, Danvers, MA, USA), anti-p-AMPK-Thr172 (1:1000; Cell Signaling), anti-mTOR (1:1000; Cell Signaling), anti-p-mTOR-Ser2448 (1:1000; Cell Signaling), ULK1 (1:1000; Cell Signaling), and p-ULK1-Ser757 (1:1000; Cell Signaling). Targeted bands were normalized to GAPDH to confirm equal protein loading. The Western blot bands were quantified using ImageJ 3.0 software.

### Immunofluorescence staining

The samples were treated with 4% paraformaldehyde for 20 min and blocked with 1% BSA and 0.2% Triton-X for 5 min. Then, they were treated with TUNEL mixture test solution (Beyotime, Shanghai, China) for 1 h at 37 °C, followed by treatment with DAPI staining solution (Boster, Wuhan, China) for 20 min at room temperature. Finally, the results were captured using a fluorescence microscope. The apoptosis index was expressed as the proportion of TUNEL-positive cells to total cardiomyocytes.

### Statistical analysis

The values in this study are expressed as the mean ± SD. All statistical analyses were performed using GraphPad Prism 6 statistical software. Statistical significance was analysed using one-way ANOVA with the Bonferroni multiple comparisons test when equal variances were assumed or Tamhane’s T2 multiple comparisons test when unequal variances were assumed. The homogeneity of variance was tested using Bartlett’s test. Statistical significance was defined as *P* < 0.05.

## Results

### Nrg4 alleviates myocardial injury in type 1 diabetic mice

**Fig. 1:**
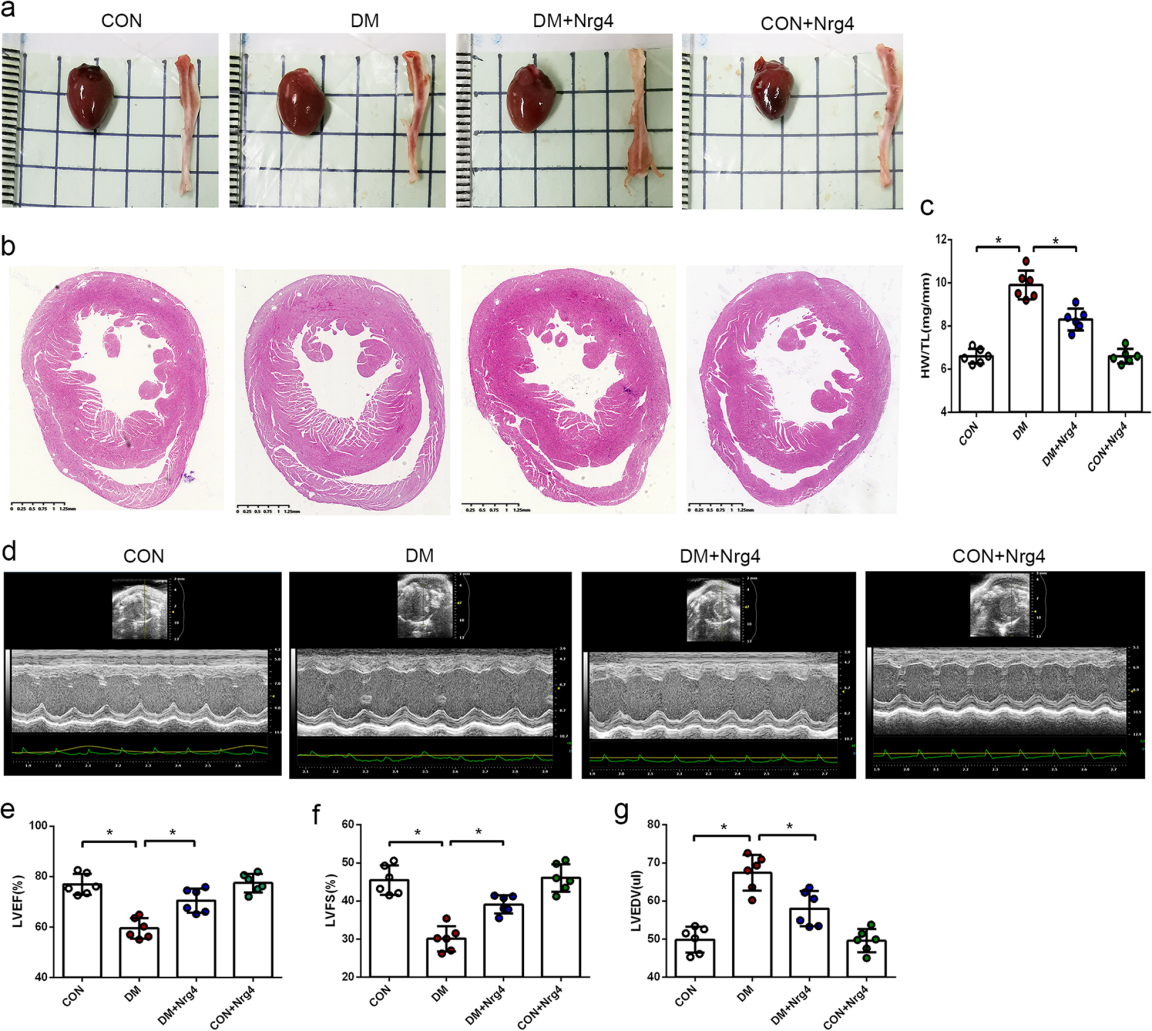
Nrg4 alleviates cardiac hypertrophy and cardiac dysfunction. **a** Representative cardiac and tibial specimens of each group. **b** Cross-sectional view of cardiac specimens from each group. **c** Ratio of heart weight to tibia length (HW/TL). **d** Representative echocardiographic images. **e** Left ventricular ejection fraction (LVEF). **f** Left ventricular fraction shortening (LVFS). **g** Left ventricular end diastolic volume (LVEDV). Data are presented as the mean ± SD (n = 6 per group). Significant differences are depicted with ^*^P < 0.05

In this study, no accidental mortality was observed during the experiment in any group. The changes in blood glucose level and body weight in each group of mice during the experiment are shown in Additional file 1: Fig. S1. Nrg4 treatment significantly prevented the development of cardiac hypertrophy and cardiac dysfunction in type 1 diabetic mice. The appearance and cross-section view of representative cardiac specimens from each group were analysed. Eight weeks after the successful establishment of the diabetes model, the heart of the DM group was significantly larger than that of the CON group, but the enlarged heart phenotype was improved in the Nrg4 group (treated with Nrg4 in the last 4 weeks) (Fig. 1a, b). The ratio of heart weight to tibial length was increased in the DM group, and Nrg4 treatment significantly ameliorated this change (Fig. 1c). Echocardiography examination also revealed that DM induced cardiac dilation with dysfunction. LVEDV was increased in the DM group compared with the CON group, whereas Nrg4 significantly inhibited the increase. LVEF and LVFS were reduced in the DM group, indicating impaired cardiac function in diabetic mice. However, treatment with Nrg4 improved the impaired heart function (Fig. 1d–g).

**Fig. 2:**
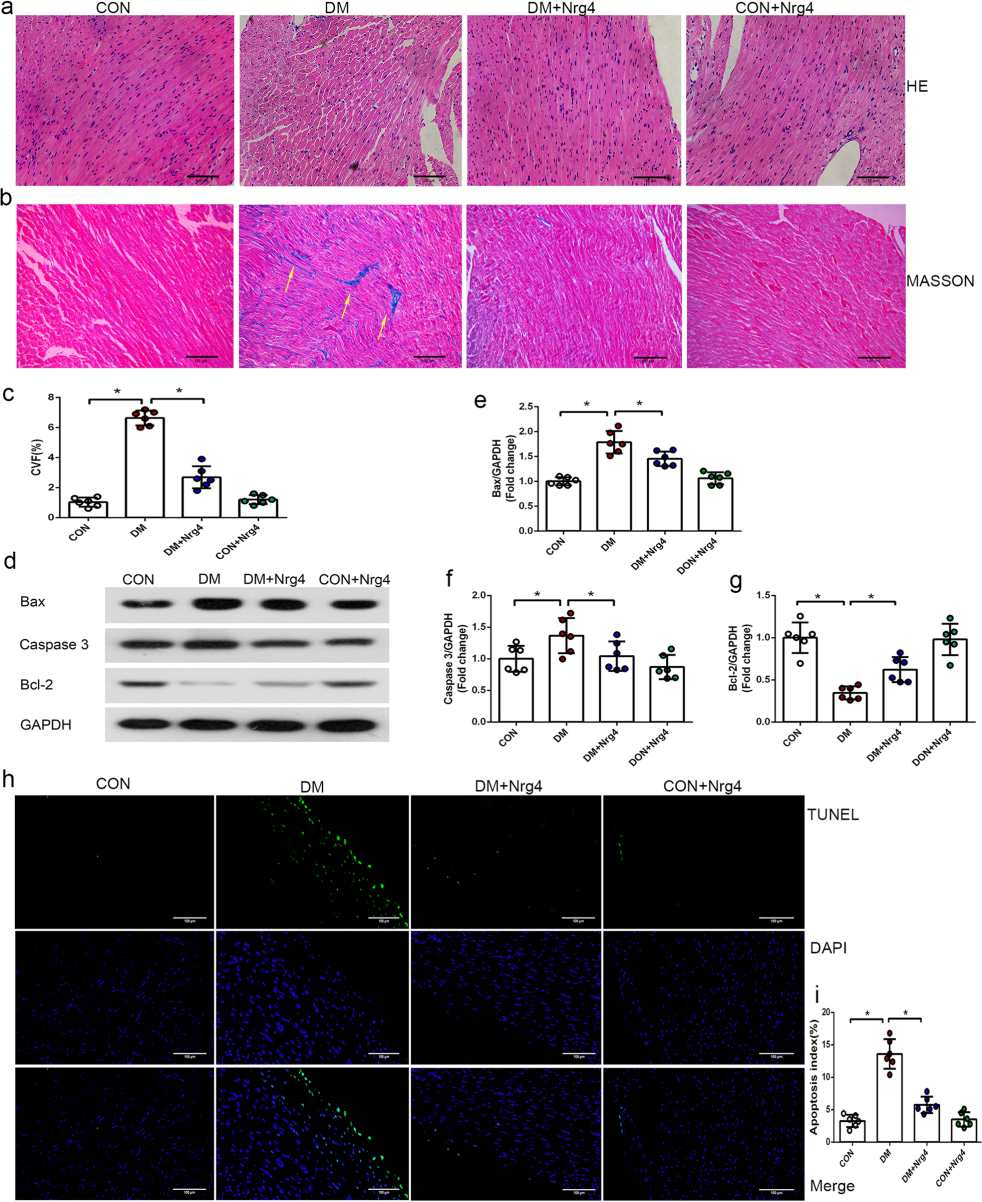
Nrg4 alleviates cardiac fibrosis and cardiomyocyte apoptosis.** a** Left ventricle stained with HE (original magnification ✕ 200). **b** Interstitial fibrosis of the left ventricles by Masson staining. The collagen fibres are stained blue (with arrows, original magnification ✕ 200). **c** Comparison of the collagen volume fraction (CVF) in each group. ** d**–**g** Western blot and quantitative analysis of apoptosis-related proteins (Bax, Caspase-3 and Bcl-2). **h** Representative immunofluorescent images with TUNEL (green) and DAPI (blue) staining (original magnification ✕ 200). **i** Comparison of the apoptosis index in each group. Data are presented as the mean ± SD (n = 6 per group). Significant differences are depicted with ^*^P < 0.05

To obtain more evidence of the beneficial effects of Nrg4 on diabetic mice, we examined the pathological structure of myocardial tissue. HE staining showed that the myocardial fibres of the CON group were arranged closely and regularly, and the nuclei were clearly visible. However, in the DM group, the cardiomyocytes presented extensive oedema and enlarged intercellular spaces, and the nuclei were not clear or were even fused. After treatment with Nrg4, the changes in cardiomyocytes were markedly relieved, and they showed a more regular arrangement (Fig. 2a). Since fibrosis has been suggested to play a key role in DM-induced cardiac pathogenesis, we quantified the fibrogenic effect of diabetes on myocardial tissue by Masson staining. Significant collagen accumulation (stained blue), predominantly in the interstitial and perivascular areas, was induced in DM mice. Treatment with Nrg4 significantly attenuated the degree of interstitial fibrosis (Fig. 2b, c).

Since cardiomyocyte apoptosis occurs as a result of DM-induced myocardial injury, we also investigated the effects of Nrg4 on the expression of apoptosis-related proteins (Fig. 2d). The level of Caspase-3 was significantly higher in the DM group than in the CON group, and Nrg4 treatment decreased the level of Caspase-3. In the DM group, Bax protein was upregulated, and Bcl-2 protein was downregulated compared with the CON group. Nrg4 intervention decreased the Bax level and increased the Bcl-2 level in the myocardium of diabetic mice (Fig. 2e–g). The TUNEL assay revealed the same changes. Myocardial apoptotic cell levels were increased in the DM group compared with the CON group, and cell death was significantly reduced after Nrg4 treatment (Fig. 2h, i).

### Autophagy is suppressed in type 1 DM, and Nrg4 can upregulate autophagy

The above results showed Nrg4-mediated cardiac protection in diabetic cardiomyopathy. We next explored the underlying mechanisms of Nrg4 intervention. Since the disruption of autophagy is essential for diabetic cardiomyopathy [34], we wondered whether Nrg4 attenuated myocardial injury by regulating autophagy. To assess the effect of this protein on autophagy, we detected the formation of autophagosomes (in vitro) and measured the expression of Beclin1, LC3-II/I and p62 (in vivo). The microtubule-associated protein LC3-I is converted into LC3-II during autophagy, and LC3-II is considered a marker of autophagosome formation. The ratio of LC3-II/LC3-I is a hallmark of autophagic activity, and Beclin1 is an activator of autophagy that is necessary for vesicle nucleation [35]. To determine whether the increase in LC3-II is due to stimulated autophagy rather than the blocked fusion of autophagosomes and lysosomes, we simultaneously measured P62 protein levels [36].

**Fig. 3:**
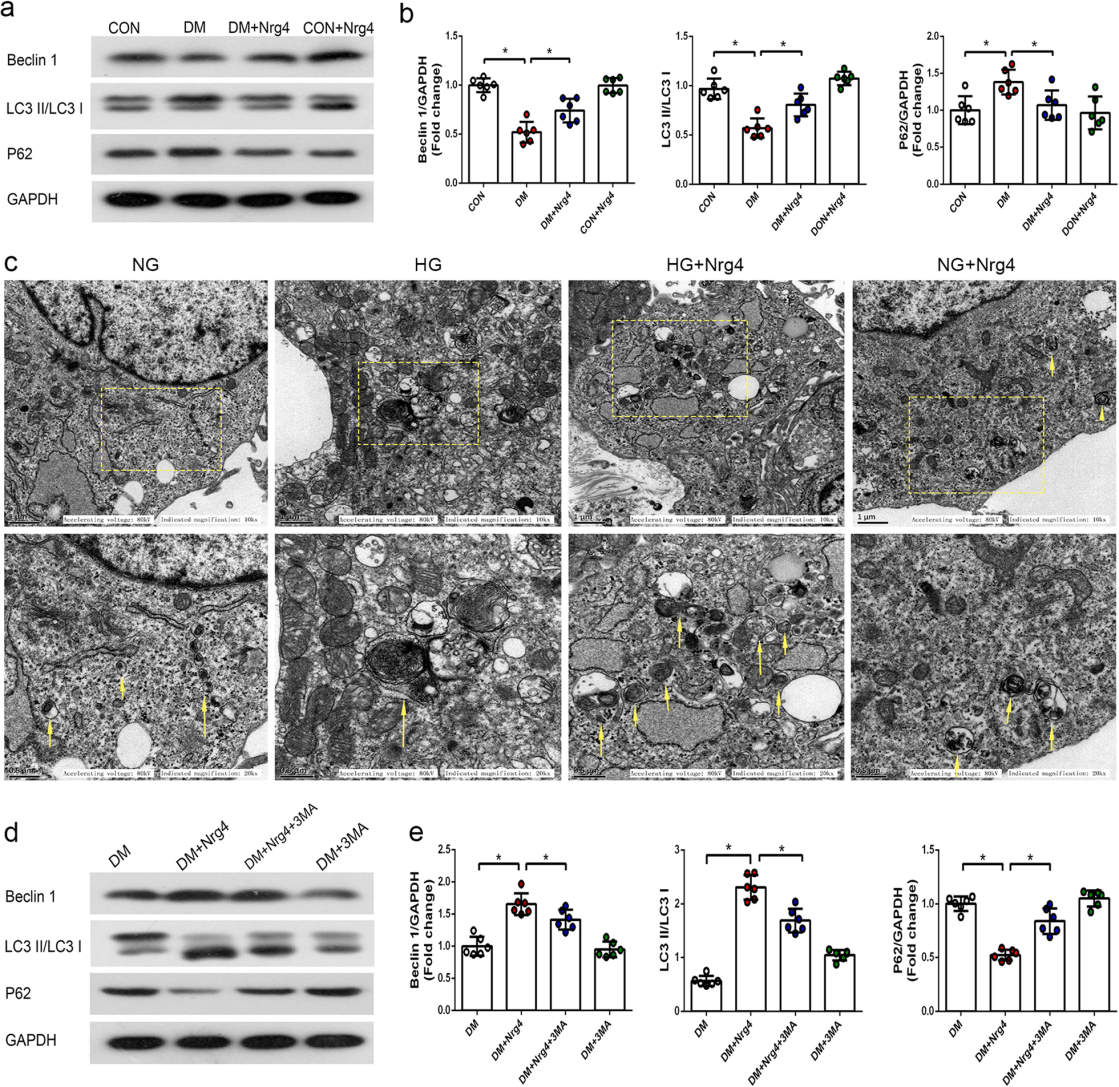
Nrg4 upregulates the suppressed autophagy in type 1 DM, and 3-MA attenuates Nrg4-upregulated autophagy. **a**, **b** Western blot and quantitative analysis of autophagy-related proteins (Beclin1, LC3-II/I and P62) in vivo. **c** Representative TEM images depicting autophagosomes in vitro, with arrows indicating autophagosomes. **d**, **e** Western blot and quantitative analysis of autophagy-related proteins in step 2 of the animal experiment. Data are presented as the mean ± SD (n = 6 per group). Significant differences are depicted with ^*^P < 0.05

Western blot analyses revealed a significant reduction in the LC3-II/I ratio and Beclin1 expression and an increase in P62 expression in the DM group compared with the CON group. Moreover, treatment with Nrg4 increased the levels of LC3-II/I and Beclin1 and decreased the protein level of P62 compared with the DM group (Fig. 3a, b). Furthermore, we confirmed the effect of Nrg4 administration on autophagy in vitro. Autophagosomes with double or multiple membrane boundaries surrounding mitochondria or other cellular organelles in the cytoplasm were examined by TEM. The results showed a significant reduction in autophagosomes in the HG group compared with the NG group, and the number of autophagosomes was increased in the HG + Nrg4 group (Fig. 3c).

### Nrg4-mediated cardiac protection is associated with the upregulation of autophagy

Our previous studies have shown that Nrg4 activated autophagy in type 1 diabetes. To determine whether the attenuation of myocardial injury by Nrg4 intervention depends on autophagy, we applied an autophagy inhibitor for reverse validation in the second step of animal experiments. As shown in the Western blot results (Fig. 3d), LC3-II/I and Beclin1 levels were significantly elevated and P62 levels were decreased by Nrg4 administration. In addition, compared with the DM + Nrg4 group, 3-MA coadministration inhibited the autophagy that was upregulated by Nrg4, as demonstrated by a decrease in the protein levels of LC3-II/I and Beclin1 and an increase in the protein expression of P62 (Fig. 3e).

**Fig. 4:**
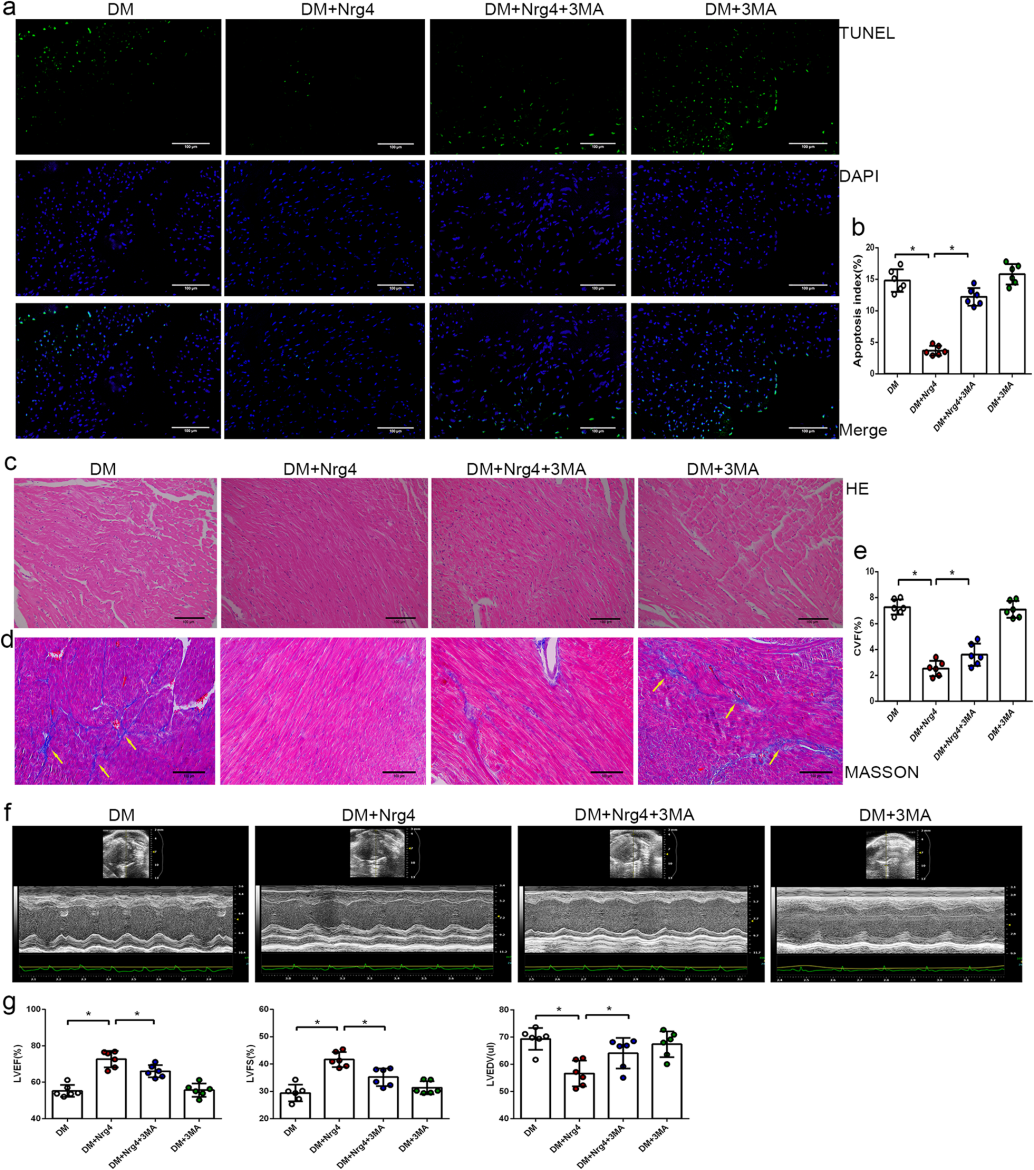
3-MA attenuates the protective effect of Nrg4 on diabetic cardiomyopathy. **a** Representative immunofluorescent images with TUNEL (green) and DAPI (blue) staining (original magnification ✕ 200). **b** Comparison of the apoptosis index in each group. **c** Stained with HE (original magnification ✕ 200). **d** Stained with Masson staining (original magnification ✕ 200). **e** Comparison of CVF in each group. **f** Representative echocardiographic images. **g** Quantitative analysis of LVEF, LVFS and LVEDV. Data are presented as the mean ± SD (n = 6 per group). Significant differences are depicted with ^*^P < 0.05

Similarly, 3-MA can also attenuate the protective effect of Nrg4 on DM-induced myocardial injury. A TUNEL assay was performed to detect cardiomyocyte apoptosis in heart tissues. Quantitative analyses revealed that the percentage of TUNEL-positive cardiomyocytes was lower in the DM + Nrg4 group than in the DM group, and the effect of Nrg4 on decreasing cell death was partly blocked by 3-MA (Fig. 4a, b). In the pathological experiment, HE staining revealed myocardial fibre disruption in DM mice, and Nrg4 treatment ameliorated the abnormality (although this effect was not as robust in the DM + Nrg4 + 3MA group) (Fig. 4c). Masson staining revealed cardiac collagen accumulation in the DM group, suggesting the induction of fibrosis. Nrg4 treatment significantly reduced cardiac fibrosis, and 3-MA inhibited the antifibrogenic effect of Nrg4 (Fig. 4d, e). In addition, heart function was markedly improved in the group of Nrg4-treated diabetic mice, as demonstrated by echocardiographic analysis. It is worth noting that this effect was attenuated when Nrg4 was coadministered with 3-MA (Fig. 4f, g). An autophagy inhibitor attenuated the Nrg4-upregulated autophagy and the protective effect on myocardial injury, indicating that Nrg4 exerts a cardioprotective effect by activating autophagy.

### Nrg4 activates autophagy via the AMPK/mTOR signalling pathway

**Fig. 5:**
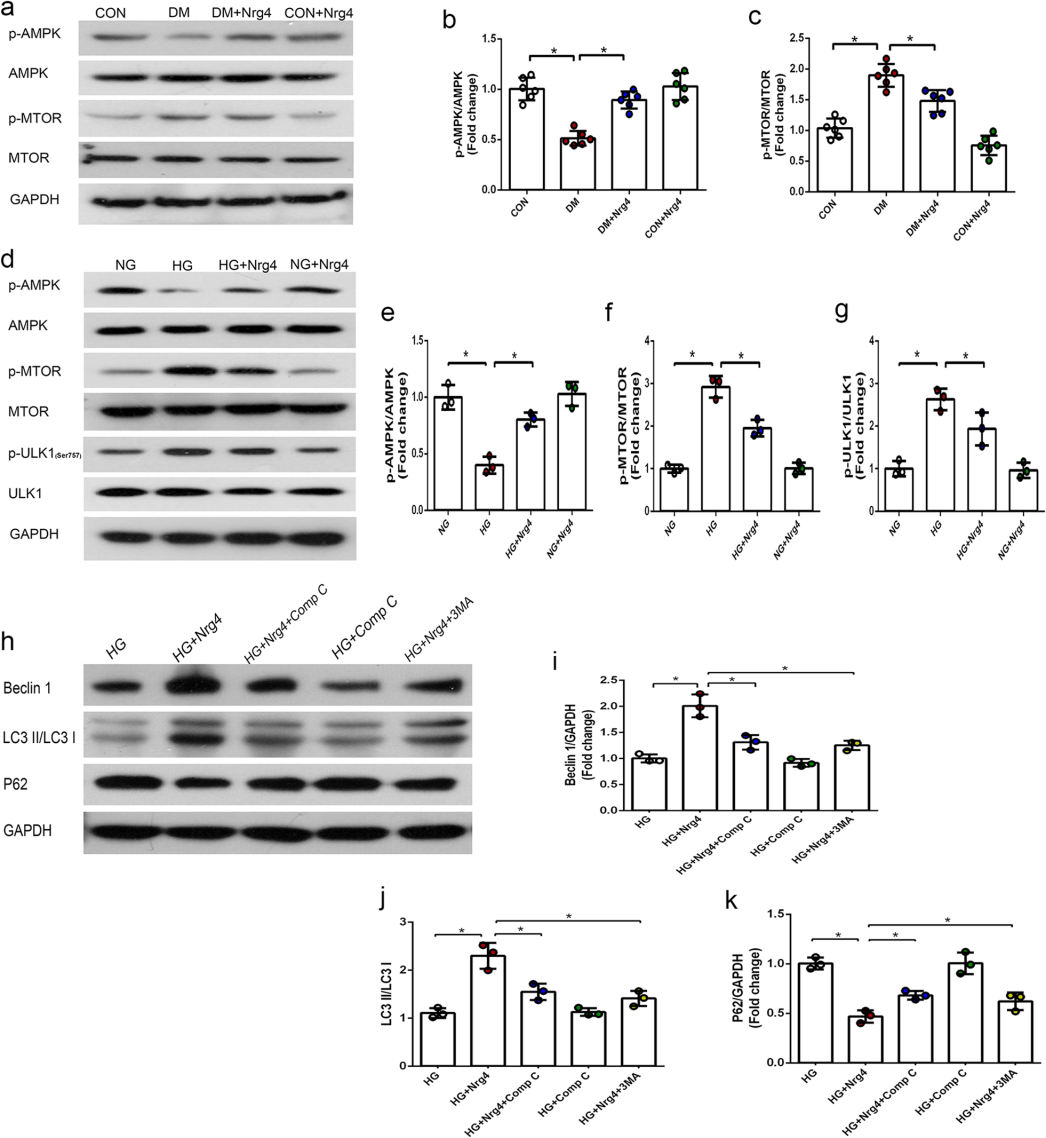
Nrg4 activates autophagy via the AMPK/mTOR signalling pathway.** a–c** Western blot and quantitative analysis of the expression of p-AMPK and p-mTOR in vivo. (n = 6 per group). **d–g** Western blot and quantitative analysis of p-AMPK, p-mTOR and p-ULK1 in vitro (n = 3 independent experiments). **h–k** Western blot and quantitative analysis of autophagy-related proteins in vitro (n = 3 independent experiments). All data are presented as the mean ± SD. Significant differences are depicted with ^*^P < 0.05

Finally, we tested the hypothesis that the cardioprotective effect of Nrg4 may be mediated by the AMPK/mTOR signalling pathway. We first explored this pathway in vivo and found that DM markedly decreased AMPK phosphorylation and increased mTOR phosphorylation. However, Nrg4 treatment partially ameliorated these changes (Fig. 5a–c). Next, we verified the activation effects of Nrg4 intervention on this signalling pathway in vitro. As expected, p-AMPK levels were elevated by Nrg4 administration compared with HG-treated cells. Subsequently, p-mTOR and p-ULK1 (a downstream signalling molecule that negatively regulates autophagy at phosphorylation Ser757) levels were decreased, indicating that Nrg4 intervention could activate the AMPK/mTOR signalling pathway (Fig. 5d–g).

### AMPK/mTOR-mediated autophagy is required for the Nrg4-induced cardioprotective effect

**Fig. 6:**
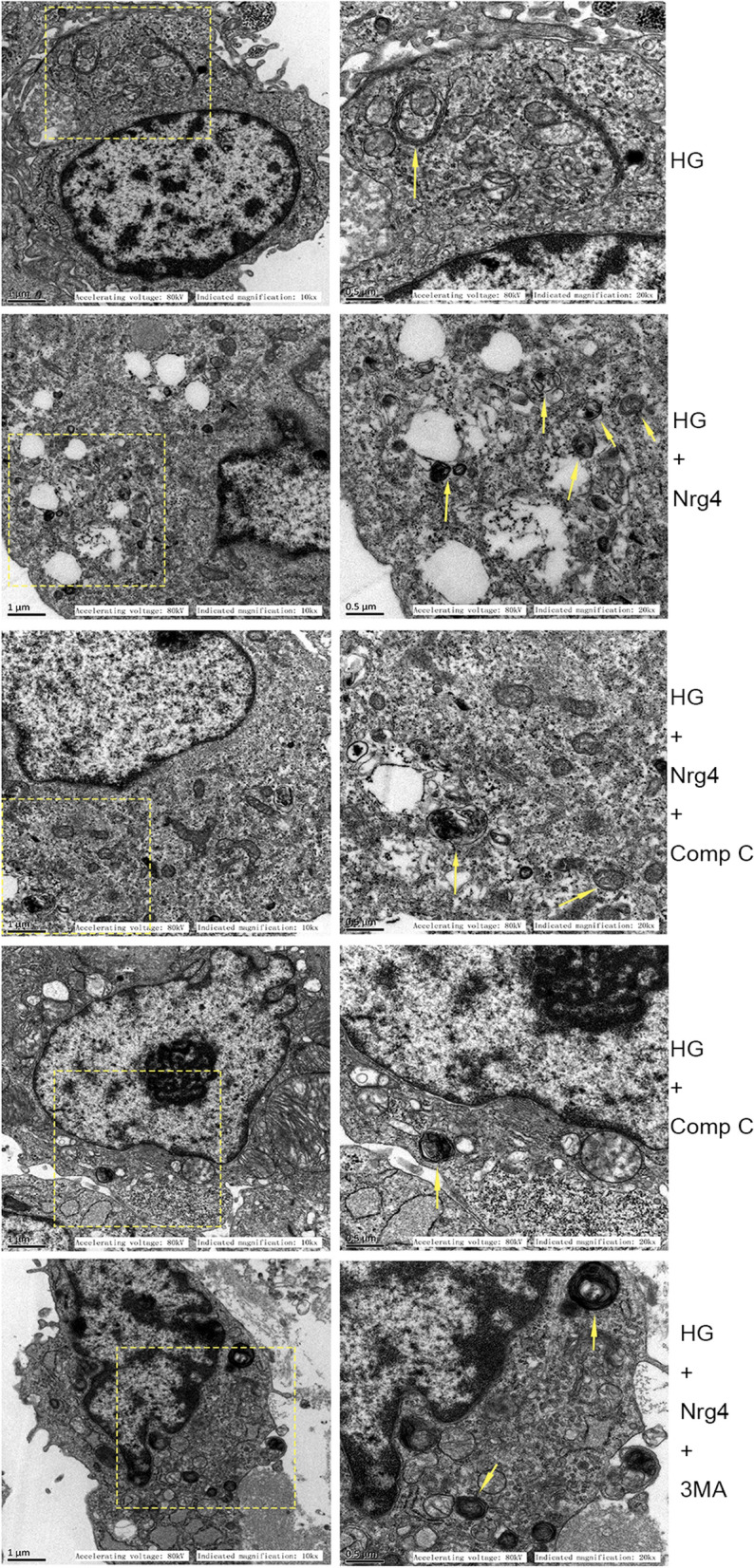
Representative TEM images depicting autophagosomes in the vitro experiment (with arrows indicating autophagosomes)

To gain insight into the relationships among Nrg4, autophagy and the AMPK/mTOR pathway, Comp C (an AMPK inhibitor) or 3-MA was coadministered with Nrg4 to high glucose-treated cardiomyocytes. We found that LC3-II/I and Beclin l levels were significantly decreased and P62 levels were markedly increased after the addition of Comp C (the same as 3-MA). These results indicate that Nrg4 activates autophagy at least partially through the AMPK/mTOR signalling pathway (Fig. 5h–k). Subsequently, we confirmed this finding by observing autophagosomes by TEM. The number of autophagosomes increased after Nrg4 treatment, while the number decreased after Comp C or 3-MA coadministration with Nrg4 (Fig. 6).

**Fig. 7:**
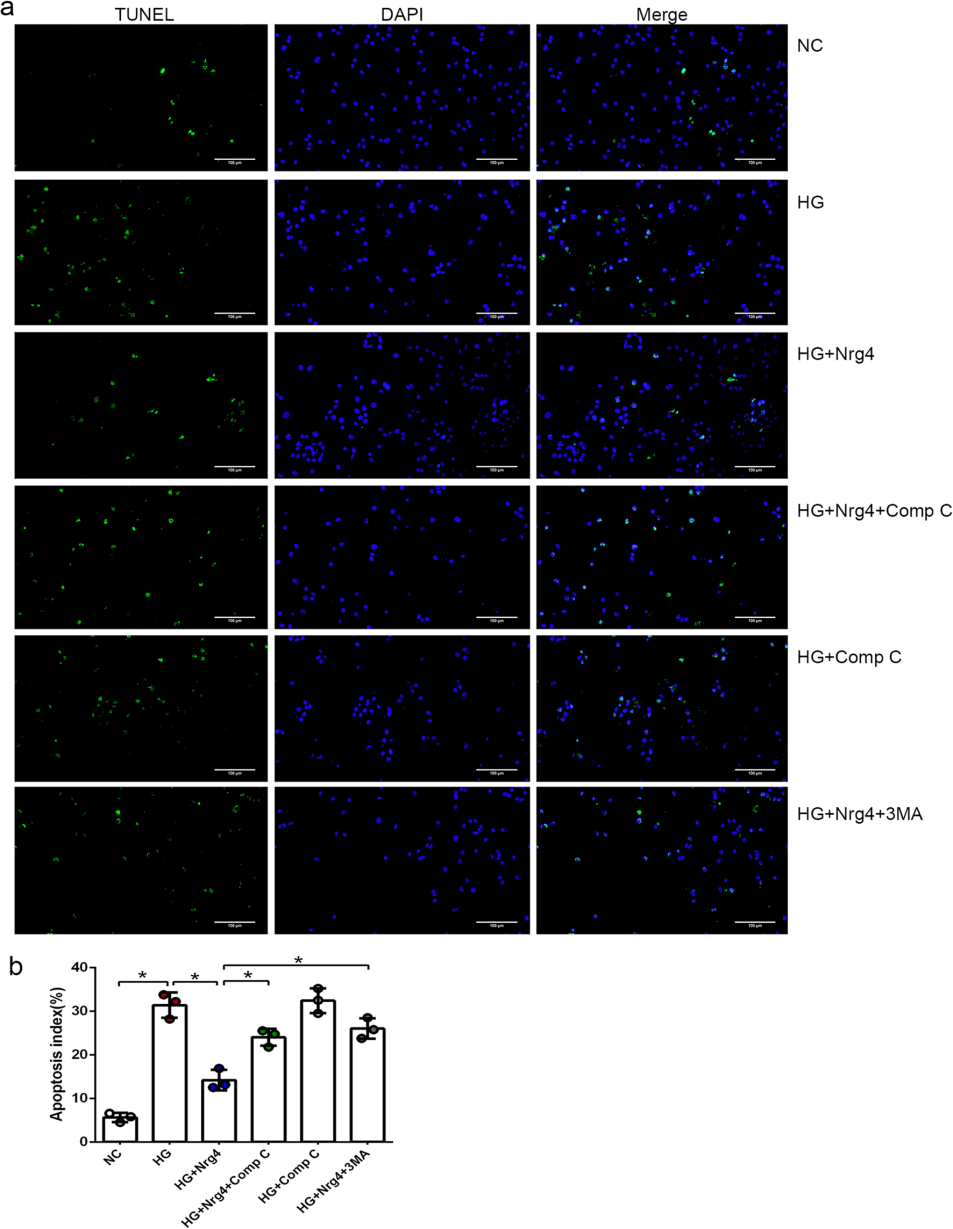
Compound C attenuates the protective effect of Nrg4 on cardiomyocyte apoptosis. **a** Representative immunofluorescent images with TUNEL in vitro (original magnification × 200). **b** Apoptosis indexes of different groups in vitro. Data are presented as the mean ± SD (n = 3 independent experiments). Significant differences are depicted with ^*^P < 0.05

Furthermore, a TUNEL assay was conducted to evaluate the effect of Nrg4 on cardiomyocyte apoptosis in vitro. The number of TUNEL-positive cells was significantly increased in the HG group compared with the NC group, and Nrg4 treatment markedly mitigated high glucose-induced apoptosis. Upon the combination of Nrg4 and Compound C or 3-MA, the protective effect of Nrg4 on apoptosis was abrogated (Fig. 7a, b). Collectively, these results indicate that the AMPK inhibitor attenuates the autophagy that is upregulated by Nrg4 and suppressed its protective effect on myocardial injury, similar to the autophagy inhibitor 3-MA.

## Discussion

The results of our study identified, for the first time, the protective effect of Nrg4 on diabetic cardiomyopathy and explored the underlying mechanisms. The major findings were as follows: (1) Nrg4 could alleviate DM-induced myocardial injury; (2) Nrg4 attenuated diabetic cardiomyopathy by upregulating autophagy in a type 1 diabetic mouse model; and (3) Nrg4 activated autophagy via the AMPK/mTOR-dependent signalling pathway.

Nrg4 is a member of the epidermal growth factor family of extracellular ligands and has been found to be highly enriched in brown adipose tissue [37]. The adipokine Nrg4 plays an important role in regulating systemic energy metabolism and is involved in the pathogenesis of obesity-associated disorders, including DM and nonalcoholic fatty liver disease [38]. Previous investigations focused on the hypothesis that Nrg4 might play roles in the development of DM and circulating Nrg4 levels might be associated with an imbalance in glucose metabolism [39, 40]. Early studies on its biological activity concentrated on the protective effects against metabolic disorders [41], insulin resistance [11, 42], hepatic steatosis [43] and tubulointerstitial fibrosis [27]. A recent study has shown that upregulation of Nrg4 gene expression can inhibit cardiomyocyte apoptosis, reverse myocardial fibrosis and play a cardioprotective role in spontaneously hypertensive rats [44]. Our study is the first to investigate whether Nrg4 has a protective effect against diabetic myocardial injury, and the results are informative. Nrg4 alleviated diabetic cardiomyopathy in various aspects: improving cardiac function, ameliorating myocardial pathological abnormalities and interstitial fibrosis, and reducing cardiomyocyte apoptosis. The results of our present study demonstrated that Nrg4 has a potential therapeutic effect on DM-induced myocardial injury.

Although the beneficial effects of Nrg4 on metabolic syndrome have been documented in previous studies, the underlying mechanism has not been fully elucidated. In the current study, we focused on autophagy, a basic catabolic mechanism for the degradation and recycling of proteins and organelles. Autophagy dysregulation has been observed in diabetic cardiomyopathy in preclinical studies [24]. However, the precise role of autophagy in this disease has yet to be established. It appears to play double roles in diabetic cardiomyopathy [45]. Both protective and pathogenic roles of autophagy in type 1 and 2 diabetes have been revealed in several studies. The role of autophagy in cardiac pathogenesis has been reported inconsistently among different types of DM [19, 21, 46, 47]. Several studies have shown increased levels of markers of autophagy in type 2 diabetic hearts, but emerging evidence from animal studies suggests that autophagy is suppressed in the heart in the presence of type 1 DM. In accordance with previous findings, our results demonstrated that autophagy was reduced in type 1 diabetic myocardium and high glucose cultured primary cardiomyocytes, as evidenced by fewer autophagosomes, decreased Beclin-1 levels and LC3-II/LC3-I ratios, and increased p62 protein expression. Moreover, we observed that Nrg4 intervention attenuated these alterations. This indicates that Nrg4 activates autophagy, which was already inhibited. In our additional experiments, the increased autophagy, as well as the protective effect on myocardial injury, was partly abolished when 3-MA was coadministered with Nrg4. These results indicate that autophagy, at least partially, mediates the improvement of myocardial injury through Nrg4 intervention. Therefore, we demonstrated that Nrg4 treatment alleviated diabetic cardiomyopathy by promoting autophagy.

**Fig. 8:**
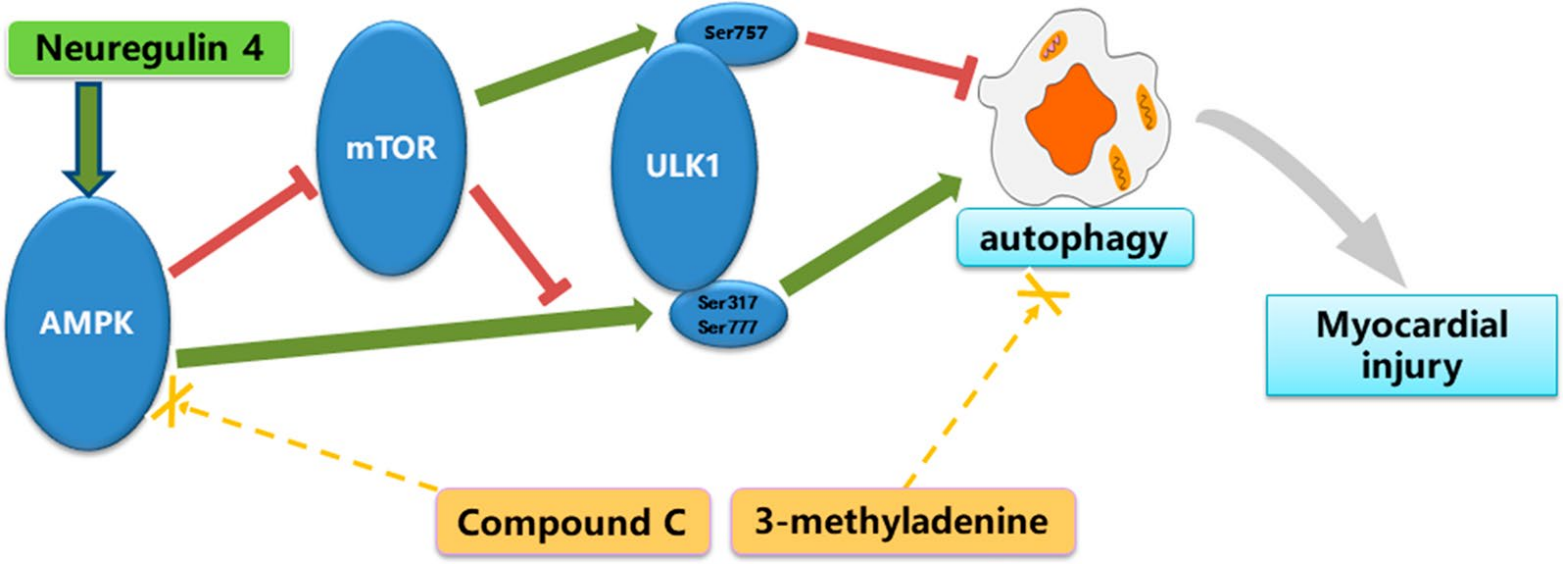
Schematic diagram of the mechanisms of autophagy induced by Nrg4 intervention

Next, we also shed light on the molecular signalling pathways that may regulate the autophagy induced by Nrg4 intervention. AMPK is a key activator of autophagy that suppresses mTOR, a negative regulator of autophagy [48]. As an autophagy-initiating kinase, ULK1 is a downstream signalling molecule in the AMPK/mTOR signalling pathway. AMPK and mTOR regulate autophagy through the coordinated phosphorylation of ULK1. This coordinated phosphorylation is important for ULK1 in autophagy induction. AMPK promotes autophagy by directly activating ULK1 through the phosphorylation of Ser317 and Ser777. mTOR suppresses autophagy via preventing ULK1 activation by phosphorylating ULK1 at Ser757 and disrupting the interaction between AMPK and ULK1 [24] (Fig. 8). Several pharmacotherapeutic agents have already been reported to protect against diabetic cardiomyopathy by triggering autophagy through AMPK activation and/or mTOR suppression [23, 49]. More importantly, Nrg4 could also activate this signalling pathway to regulate autophagy in hepatocytes [25]. In our study, we demonstrated that Nrg4 intervention could activate AMPK, inhibit mTOR, and thereby induce autophagy both in vivo and in vitro. In cardiomyocytes treated with HG, the phosphorylation of AMPK decreased, and the phosphorylation of mTOR increased. This was accompanied an increase in the phosphorylation of ULK1_Ser757_. AMPK activates ULK1 by phosphorylating Ser317 and Ser777. The activation of ULK1 through the phosphorylation of Ser317/Ser777 has a critical role in autophagy induction. The elevated mTOR activity disrupts the interaction between AMPK and ULK1 through the phosphorylation of ULK1 at Ser757, thereby preventing ULK1 activation [50]. All these changes were subsequently mitigated after Nrg4 intervention. Furthermore, the simultaneous treatment of Nrg4 and compound C attenuated the autophagy induced by Nrg4 and suppressed its protective effect on cardiomyocytes. These findings indicate that AMPK/mTOR-dependent autophagy was necessary for protecting HG-induced myocardial injury. Together, our results confirm that the AMPK/mTOR signalling cascade participates in autophagy induction through Nrg4 intervention.

Collectively, our study provides new mechanistic insight into the alleviation of diabetic cardiomyopathy through autophagy intervention by Nrg4, a potential pharmacotherapeutic agent. Whether Nrg4 may become a clinically relevant approach for the treatment of diabetic cardiomyopathy needs to be further explored.

## Conclusion

Neuregulin-4 attenuates diabetic cardiomyopathy by regulating autophagy via the AMPK/mTOR signalling pathway in type 1 diabetic mice. Targeting autophagy through Nrg4 intervention may represent a therapeutic strategy for diabetic heart conditions and for the clinical management of DM.

## Supplementary Information

**Additional file 1: Figure S1.**Changes in the metabolic indicators in each group of mice during the experiment. **a** In step 1 of the animal experiment, the blood glucose level of diabetic mice increased significantly after modelling. It then showed a downward trend in the later stage of the experiment but remained higher than 16.7 mmol/L (random blood glucose). **b** Changes in the body weight of mice in step 1 of the animal experiment. **c** Changes in the blood glucose level and body weight after the establishment of diabetes mouse model in step 2 of the animal experiment. The changes in blood glucose level were the same as those in step 1 animal experiment.

## Data Availability

The datasets used and/or analysed during the current study are available from the corresponding author upon reasonable request.

## Abbreviations

DM: Diabetes mellitus
Nrg4: Neuregulin-4
3-MA: 3-methyladenine
DMEM: Dulbecco’s modified Eagle’s medium
NG: Normal glucose
HG: High glucose
Comp C: Compound C
LVEDD: Left ventricular end-diastolic diameter
LVESD: Left ventricular end-systolic diameter
LVEDV: Left ventricular end-diastolic volume
LVEF: Left ventricular ejection fraction
LVFS: Left ventricular fractional shortening
HE: Haematoxylin & eosin
CVF: Collagen volume fraction
TEM: Transmission electron microscopy

## References

[1] Tate M, Grieve DJ, Ritchie RH (2017). Are targeted therapies for diabetic cardiomyopathy on the horizon. Clin Sci (Lond).

[2] Kenny HC, Abel ED (2019). Heart failure in type 2 diabetes mellitus. Circ Res.

[3] Jia G, Hill MA, Sowers JR (2018). Diabetic cardiomyopathy: an update of mechanisms contributing to this clinical entity. Circ Res.

[4] Seferović PM, Paulus WJ (2015). Clinical diabetic cardiomyopathy: a two-faced disease with restrictive and dilated phenotypes. Eur Heart J.

[5] Wang L, Cai Y, Jian L, Cheung CW, Zhang L, Xia Z (2021). Impact of peroxisome proliferator-activated receptor-α on diabetic cardiomyopathy. Cardiovasc Diabetol.

[6] Ghosh N, Katare R (2018). Molecular mechanism of diabetic cardiomyopathy and modulation of microRNA function by synthetic oligonucleotides. Cardiovasc Diabetol.

[7] Sato T, Minatsuki S (2019). Neuregulin-4, an adipokine, as a residual risk factor of atherosclerotic coronary artery disease. Int Heart J.

[8] Liberale L, Montecucco F (2020). Adipocytokines and cardiovascular diseases: putative role of Neuregulin 4. Eur J Clin Invest..

[9] Chen Z, Wang GX, Ma SL, Jung DY, Ha H, Altamimi T, Zhao XY, Guo L, Zhang P, Hu CR (2017). Nrg4 promotes fuel oxidation and a healthy adipokine profile to ameliorate diet-induced metabolic disorders. Mol Metab.

[10] Wang GX, Zhao XY, Meng ZX, Kern M, Dietrich A, Chen Z, Cozacov Z, Zhou D, Okunade AL, Su X (2014). The brown fat-enriched secreted factor Nrg4 preserves metabolic homeostasis through attenuation of hepatic lipogenesis. Nat Med.

[11] Díaz-Sáez F, Blanco-Sinfreu C, Archilla-Ortega A, Sebastian D, Romero M, Hernández-Alvarez MI, Mora S, Testar X, Ricart W, Fernández-Real JM (2021). Neuregulin 4 downregulation induces insulin resistance in 3T3-L1 Adipocytes through inflammation and autophagic degradation of GLUT4 vesicles. Int J Mol Sci.

[12] Wang W, Zhang Y, Yang C, Wang Y, Shen J, Shi M, Wang B (2019). Transplantation of neuregulin 4-overexpressing adipose-derived mesenchymal stem cells ameliorates insulin resistance by attenuating hepatic steatosis. Exp Biol Med (Maywood).

[13] Yan P, Xu Y, Zhang Z, Gao C, Zhu J, Li H, Wan Q (2019). Decreased plasma neuregulin 4 levels are associated with peripheral neuropathy in Chinese patients with newly diagnosed type 2 diabetes: a cross-sectional study. Cytokine.

[14] Yan P, Xu Y, Wan Q, Feng J, Li H, Yang J, Zhong H, Zhang Z (2018). Plasma neuregulin 4 levels are associated with metabolic syndrome in patients newly diagnosed with type 2 diabetes mellitus. Dis Markers.

[15] Yan P, Zhang Z, Miao Y, Xu Y, Zhu J, Wan Q (2020). Changes of circulating neuregulin 4 and its relationship with 25-hydroxy vitamin D and other diabetic vascular complications in patients with diabetic peripheral neuropathy. Diabetol Metab Syndr.

[16] Kocak MZ, Aktas G, Atak BM, Duman TT, Yis OM, Erkus E, Savli H (2020). Is Neuregulin-4 a predictive marker of microvascular complications in type 2 diabetes mellitus. Eur J Clin Invest.

[17] Mizushima N, White E, Rubinsztein DC (2021). Breakthroughs and bottlenecks in autophagy research. Trends Mol Med.

[18] Zech A, Singh SR, Schlossarek S, Carrier L (2020). Autophagy in cardiomyopathies. Biochim Biophys Acta Mol Cell Res.

[19] Wu QQ, Liu C, Cai Z, Xie Q, Hu T, Duan M, Wu H, Yuan Y, Tang Q (2020). High-mobility group AT-hook 1 promotes cardiac dysfunction in diabetic cardiomyopathy via autophagy inhibition. Cell Death Dis.

[20] Ni T, Lin N, Lu W, Sun Z, Lin H, Chi J, Guo H (2020). Dihydromyricetin prevents diabetic cardiomyopathy via miR-34a suppression by activating autophagy. Cardiovasc Drugs Ther.

[21] Li X, Ke X, Li Z, Li B (2019). Vaspin prevents myocardial injury in rats model of diabetic cardiomyopathy by enhancing autophagy and inhibiting inflammation. Biochem Biophys Res Commun.

[22] Yang F, Qin Y, Wang Y, Meng S, Xian H, Che H, Lv J, Li Y, Yu Y, Bai Y (2019). Metformin inhibits the NLRP3 inflammasome via AMPK/mTOR-dependent effects in diabetic cardiomyopathy. Int J Biol Sci.

[23] Zhang Y, Ling Y, Yang L, Cheng Y, Yang P, Song X, Tang H, Zhong Y, Tang L, He S (2017). Liraglutide relieves myocardial damage by promoting autophagy via AMPK-mTOR signaling pathway in zucker diabetic fatty rat. Mol Cell Endocrinol.

[24] Dewanjee S, Vallamkondu J, Kalra RS, John A, Reddy PH, Kandimalla R (2021). Autophagy in the diabetic heart: a potential pharmacotherapeutic target in diabetic cardiomyopathy. Ageing Res Rev.

[25] Zhu B, Mei W, Jiao T, Yang S, Xu X, Yu H, Ding Y, Guo S, Meng B, Zhao L (2020). Neuregulin 4 alleviates hepatic steatosis via activating AMPK/mTOR-mediated autophagy in aged mice fed a high fat diet. Eur J Pharmacol.

[26] Wu B, Lin J, Luo J, Han D, Fan M, Guo T, Tao L, Yuan M, Yi F (2017). Dihydromyricetin protects against diabetic cardiomyopathy in streptozotocin-induced diabetic mice. Biomed Res Int.

[27] Shi J, Xu W, Zheng R, Miao H, Hu Q (2019). Neuregulin 4 attenuate tubulointerstitial fibrosis and advanced glycosylation end products accumulation in diabetic nephropathy rats via regulating TNF-R1 signaling. Am J Transl Res.

[28] Li R, Guo E, Yang J, Li A, Yang Y, Liu S, Liu A, Jiang X (2017). 1,25(OH)(2) D(3) attenuates hepatic steatosis by inducing autophagy in mice. Obes (Silver Spring).

[29] Jentzsch C, Leierseder S, Loyer X, Flohrschütz I, Sassi Y, Hartmann D, Thum T, Laggerbauer B, Engelhardt S (2012). A phenotypic screen to identify hypertrophy-modulating microRNAs in primary cardiomyocytes. J Mol Cell Cardiol.

[30] Wang L, Shen M, Guo X, Wang B, Xia Y, Wang N, Zhang Q, Jia L, Wang X (2017). Volume-sensitive outwardly rectifying chloride channel blockers protect against high glucose-induced apoptosis of cardiomyocytes via autophagy activation. Sci Rep.

[31] Guo S, Meng XW, Yang XS, Liu XF, Ou-Yang CH, Liu C (2018). Curcumin administration suppresses collagen synthesis in the hearts of rats with experimental diabetes. Acta Pharmacol Sin.

[32] Teichholz LE, Kreulen T, Herman MV, Gorlin R (1976). Problems in echocardiographic volume determinations: echocardiographic-angiographic correlations in the presence of absence of asynergy. Am J Cardiol.

[33] Ding Y, Jiang H, Meng B, Zhu B, Yu X, Xiang G (2019). Sweroside-mediated mTORC1 hyperactivation in bone marrow mesenchymal stem cells promotes osteogenic differentiation. J Cell Biochem.

[34] Du J, Liu Y, Fu J (2020). Autophagy, myocarditis, and cardiomyopathy. Adv Exp Med Biol.

[35] Sun L, Zhang W (2021). Preconditioning of mesenchymal stem cells with ghrelin exerts superior cardioprotection in aged heart through boosting mitochondrial function and autophagy flux. Eur J Pharmacol.

[36] Fernández ÁF (2018). Autophagy and proteases: basic study of the autophagic flux by Western blot. Methods Mol Biol.

[37] Gavaldà-Navarro A, Villarroya J, Cereijo R, Giralt M, Villarroya F (2022). The endocrine role of brown adipose tissue: an update on actors and actions. Rev Endocr Metab Disord.

[38] De Munck T, Boesch M, Verhaegh P, Masclee A, Jonkers D, van Pelt JF, du Plessis J, Korf H, Nevens F, Koek GH (2021). Is there a role for neuregulin 4 in human nonalcoholic fatty liver disease. PLoS ONE.

[39] Li M, Chen Y, Jiang J, Lu Y, Song Z, Zhang S, Sun C, Ying H, Fan X, Song Y (2019). Elevated serum neuregulin 4 levels in patients with hyperthyroidism. Endocr Connect.

[40] Blüher M (2019). Neuregulin 4: a “hotline” between brown fat and liver. Obes (Silver Spring).

[41] Li Y, Jin L, Jiang F, Yan J, Lu Y, Yang Q, Zhang Y, Zhang H, Yu H, Zhang Y (2021). Mutations of NRG4 contribute to the pathogenesis of nonalcoholic fatty liver disease and related metabolic disorders. Diabetes.

[42] Yang F, Zhou N, Zhu X, Min C, Zhou W, Li X (2021). n-3 PUFAs protect against adiposity and fatty liver by promoting browning in postnatally overfed male rats: a role for NRG4. J Nutr Biochem.

[43] Tutunchi H, Mobasseri M, Aghamohammadzadeh N, Hooshyar J, Naeini F, Najafipour F (2021). Serum neuregulin 4 (NRG-4) level and non-alcoholic fatty liver disease (NAFLD): a case-control study. Int J Clin Pract.

[44] Yang Z, Yaling W, Tao L, Mingyue R, Yongjun L (2022). Cardioprotective effect of NRG-4 gene expression on spontaneous hypertension rats and its mechanism through mediating the activation of ErbB signaling pathway. Cell Mol Biol (Noisy-le-grand).

[45] Xu T, Ding W, Ji X, Ao X, Liu Y, Yu W, Wang J (2019). Oxidative stress in cell death and cardiovascular diseases. Oxid Med Cell Longev.

[46] Packer M (2020). SGLT2 inhibitors produce cardiorenal benefits by promoting adaptive cellular reprogramming to induce a state of fasting mimicry: a paradigm shift in understanding their mechanism of action. Diabetes Care.

[47] Packer M (2020). Autophagy-dependent and -independent modulation of oxidative and organellar stress in the diabetic heart by glucose-lowering drugs. Cardiovasc Diabetol.

[48] Escobar KA, Cole NH, Mermier CM, VanDusseldorp TA (2019). Autophagy and aging: maintaining the proteome through exercise and caloric restriction. Aging Cell.

[49] Lin C, Zhang M, Zhang Y, Yang K, Hu J, Si R, Zhang G, Gao B, Li X, Xu C (2017). Helix B surface peptide attenuates diabetic cardiomyopathy via AMPK-dependent autophagy. Biochem Biophys Res Commun.

[50] Kim J, Kundu M, Viollet B, Guan KL (2011). AMPK and mTOR regulate autophagy through direct phosphorylation of Ulk1. Nat Cell Biol.

