# Supplementary figures and images for: Neuregulin-4 attenuates diabetic cardiomyopathy by regulating autophagy via the AMPK/mTOR signalling pathway
Source: Cardiovasc Diabetol. 2022 Oct 11;21:205. doi: 10.1186/s12933-022-01643-0 (PMC9554973; doi:10.1186/s12933-022-01643-0)

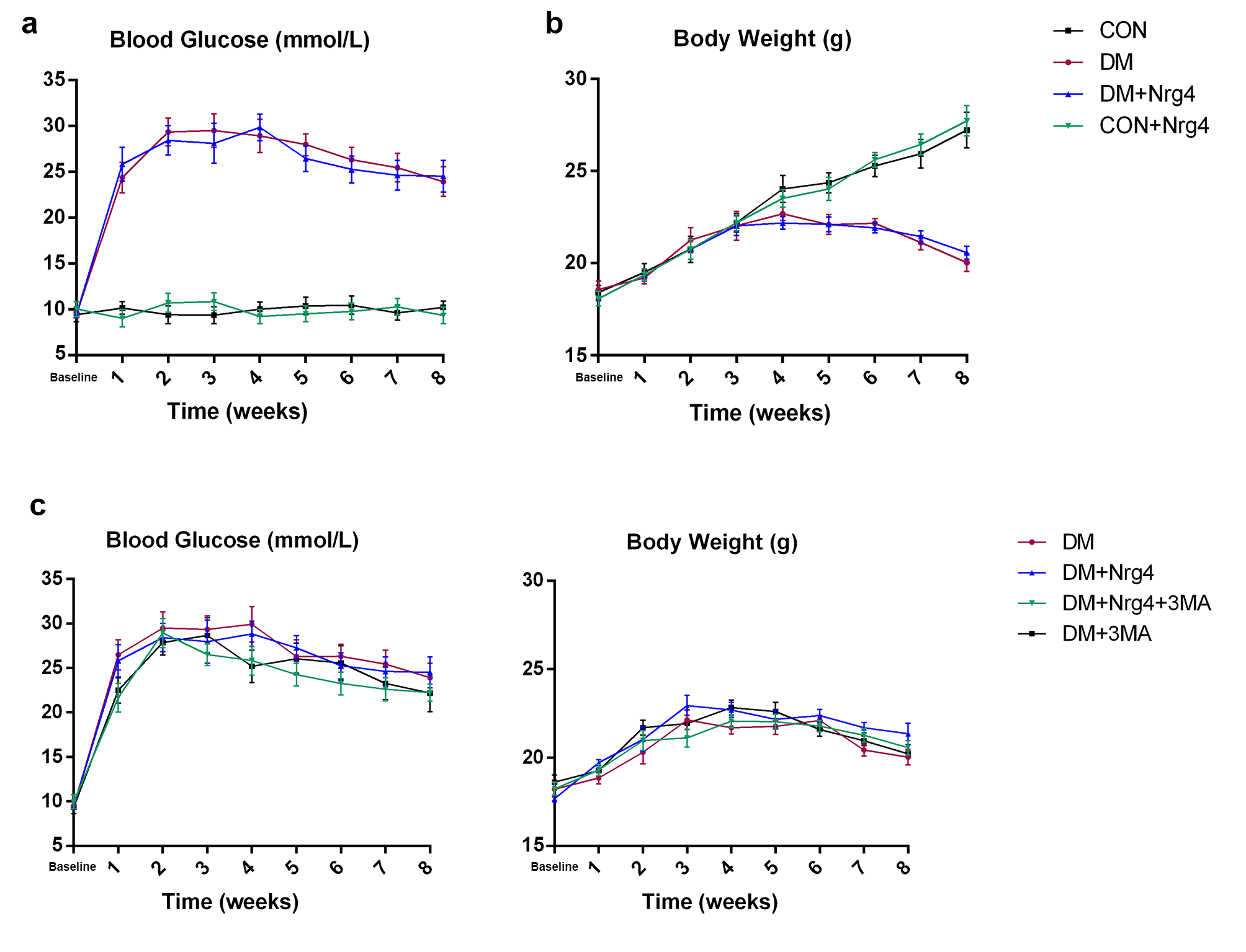

Supplement: Supplementary file 1 — Additional file 1: Figure S1.Changes in the metabolic indicators in each group of mice during the experiment. a In step 1 of the animal experiment, the blood glucose level of diabetic mice increased significantly after modelling. It then showed a downward trend in the later stage of the experiment but remained higher than 16.7 mmol/L (random blood glucose). b Changes in the body weight of mice in step 1 of the animal experiment. c Changes in the blood glucose level and body weight after the establishment of diabetes mouse model in step 2 of the animal experiment. The changes in blood glucose level were the same as those in step 1 animal experiment. [file 12933_2022_1643_MOESM1_ESM.tif]
